# Evaluating the Typical Day-to-Day Variability of WHOOP-Derived Heart Rate Variability in Olympic Water Polo Athletes

**DOI:** 10.3390/s22186723

**Published:** 2022-09-06

**Authors:** Clint R. Bellenger, Dean Miller, Shona L. Halson, Gregory D. Roach, Michael Maclennan, Charli Sargent

**Affiliations:** 1 Alliance for Research in Exercise, Nutrition and Activity (ARENA), Allied Health and Human Performance, University of South Australia, Adelaide 5000, Australia; 2 The Appleton Institute for Behavioural Science, Central Queensland University, Adelaide 5034, Australia; 3 School of Behavioural and Health Sciences, Australian Catholic University, Brisbane 4014, Australia; 4 Water Polo Australia, Sydney Olympic Park, Sydney 2127, Australia

**Keywords:** autonomic nervous system, reliability, photoplethysmography, readiness to perform, coefficient of variation

## Abstract

Heart rate (HR) and HR variability (HRV) can be used to infer readiness to perform exercise in athletic populations. Advancements in the photoplethysmography technology of wearable devices such as WHOOP allow for the frequent and convenient measurement of HR and HRV, and therefore enhanced application in athletes. However, it is important that the reliability of such technology is acceptable prior to its application in practical settings. Eleven elite male water polo players (age 28.8 ± 5.3 years [mean ± standard deviation]; height 190.3 ± 3.8 cm; body mass 95.0 ± 6.9 kg; international matches 117.9 ± 92.1) collected their HR and HRV daily via a WHOOP strap (WHOOP 3.0, CB Rank, Boston, MA, USA) over 16 weeks ahead of the 2021 Tokyo Olympic Games. The WHOOP strap quantified HR and HRV via wrist-based photoplethysmography during overnight sleep periods. The weekly (i.e., 7-day) coefficient of variation in lnRMSSD (lnRMSSD_CV_) and HR (HR_CV_) was calculated as a measure of day-to-day variability in lnRMSSD and HR, and presented as a mean of the entire recording period. The mean weekly lnRMSSD_CV_ and HR_CV_ over the 16-week period was 5.4 ± 0.7% (mean ± 95% confidence intervals) and 7.6 ± 1.3%, respectively. The day-to-day variability in WHOOP-derived lnRMSSD and HR is within or below the range of day-to-day variability in alternative lnRMSSD (~3–13%) and HR (~10–11%) assessment protocols, indicating that the assessment of HR and HRV by WHOOP does not introduce any more variability than that which is naturally present in these variables.

## 1. Introduction

The accurate assessment of readiness to perform exercise in athletes is important since it facilitates subtle manipulations in training load to optimise physiological adaptation and subsequent exercise performance [1]. For example, the accurate assessment of excessive fatigue during training periods allows coaches and sport science practitioners to prioritise recovery, thereby avoiding the undesired training states of non-functional overreaching and overtraining [1].

Assessment of autonomic nervous system function by heart rate (HR) and HR variability (HRV) are popular and sensitive measures of readiness to perform exercise in athletes [2]. Specifically, HRV is a sensitive marker of the physiological response to acute training sessions [3,4], and a sensitive marker of improvements [5] and decrements [6,7,8] in exercise performance following longitudinal training programs. Consequently, endurance training guided exclusively by HRV assessment has been shown to effectively improve exercise performance [9,10,11].

The quantification of typical day-to-day variability in any physiological variable (including HR and HRV) is an important process in the application of this variable for inferring readiness to perform exercise. Day-to-day variability concerns the reproducibility of an observed value when a measurement is repeated [12], and is important to quantify as it allows sport and exercise science practitioners to separate a “true” change in a variable of interest from the inherent “noise” in the variable. Regarding HRV assessment, typical day-to-day variability in the natural logarithm of the root mean square of successive R wave to R wave differences (lnRMSSD) ranges between 3 and 13% measured via coefficient of variation [2,13,14,15,16,17,18,19]. The range in variability reported is likely attributable to assessment nuances, such as the timing of assessment (i.e., morning waking versus nocturnal), posture (i.e., supine versus sitting versus standing), recording device, and the training load applied to the athletes/participants at the time of assessment (i.e., no training/minimal training versus typical/baseline training versus overload training, etc.). Additionally, the typical day-to-day variability in HR is ~10–11% [19,20].

Technological advancements in wearable HR monitor technology have facilitated a number of novel recording devices for quantifying HR and HRV. WHOOP is one such recording device, with several assessment nuances. Specifically, the WHOOP3.0 unit quantifies HR and HRV via wrist-based photoplethysmography during overnight sleep periods [21]. The validity of WHOOP3.0-derived HR and HRV, and its determination of sleep, has been previously demonstrated [22,23], however the typical day-to-day variability (i.e., reliability) in WHOOP3.0-derived HR and HRV has yet to be determined.

Consequently, given the novelty of WHOOP and its assessment nuances for quantifying HR and HRV, the primary aim of this study was to determine the typical day-to-day variability in WHOOP3.0-derived HR and HRV in Olympic water polo athletes during a period of habitual training. The impact of training load on day-to-day variability in WHOOP3.0-derived HR and HRV was also of interest, with the specific focus of determining day-to-day variability during weeks corresponding to typical or baseline training load.

## 2. Materials and Methods

### 2.1. Experimental Overview and Participants

This study concerns a retrospective analysis of data collected from 11 elite water polo athletes (age 28.8 ± 5.3 years [mean ± standard deviation]; height 190.3 ± 3.8 cm; body mass 95.0 ± 6.9 kg; international matches 117.9 ± 92.1) during a 16-week period of routine training in preparation for the 2021 Tokyo Olympic Games. The sample size was fixed given the retrospective analysis study design. Athletes provided written informed consent for the inclusion of their de-identified data, and the University of South Australia’s Human Research Ethics Committee approved the retrospective analysis of these de-identified data.

### 2.2. Data Collection and Analysis

Athletes collected their HR and HRV (i.e., RMSSD) daily via WHOOP strap (WHOOP 3.0, CB Rank, Boston, MA, USA) use during overnight sleep periods [21]. WHOOP3.0-derived HR and HRV data from the 16-week recording period were extracted into a customised Microsoft Excel spreadsheet for analysis.

Natural logarithm transformation of RMSSD data (i.e., lnRMSSD) were performed to reduce bias from heteroscedasticity [24], as has become standard practice for the longitudinal monitoring of training status by HRV [2]. For each 7-day period (i.e., Monday to Sunday) during the 16-week recording period, a coefficient of variation (CV%; 7-day standard deviation as a percentage of 7-day mean) was calculated for each athlete as a measure of day-to-day variability in lnRMSSD (lnRMSSD_CV_) and HR (HR_CV_). A 7-day CV% has previously been used to quantify day-to-day variability in HRV [13,14,16,17]. Additionally, given that 7-day averages of HRV have been advocated in the longitudinal monitoring of HRV [6,8,14,25,26], it is intuitive that the day-to-day variability in HRV (and HR) be quantified over a 7-day period. To account for compliance issues in data collected in the routine training environment, a minimum of three measurements in any 7-day period was required for valid calculation [14]. The weekly values of lnRMSSD_CV_ and HR_CV_ reflect the range of day-to-day variability in WHOOP3.0-derived HRV and HR over the 16-week period of habitual training. Mean lnRMSSD_CV_ and mean HR_CV_ were also calculated from the weekly values to reflect the mean day-to-day variability in WHOOP3.0-derived HRV and HR over the 16-week period of habitual training.

To contextualise the weekly values of lnRMSSD_CV_ and HR_CV_ by training load, daily training load was quantified via WHOOP’s daily “Strain”, which measures “total cardiovascular load” on a proprietary scale of 0 to 21 [27]. For each week (i.e., Monday to Sunday) during the 16-week recording period, mean daily Strain was calculated for each athlete to reflect weekly training load. Mean weekly training load was then calculated for each athlete over the entire 16-week recording period. Subsequently, individual weekly training loads were calculated as a percentage of the 16-week mean training load, such that each training week for each athlete could be presented as a percentage of mean weekly training load during the 16-week recording period.

Finally, to determine the day-to-day variability in WHOOP3.0-derived HR and HRV during a typical/baseline training load, and to compare this load to training loads below and above this typical/baseline training load, percent weekly training load was categorised to the following loads: ≤85% (*n* = 22 data points); 85–95% (*n* = 45 data points); 95–105% (*n* = 49 data points); 105–115% (*n* = 35 data points); >115% (*n* = 25 data points), and mean weekly lnRMSSD_CV_ and HR_CV_ were calculated for each category. The typical/baseline training load was considered to be 95–105% of the mean 16-week training load.

## 3. Results

**Figure 1:**
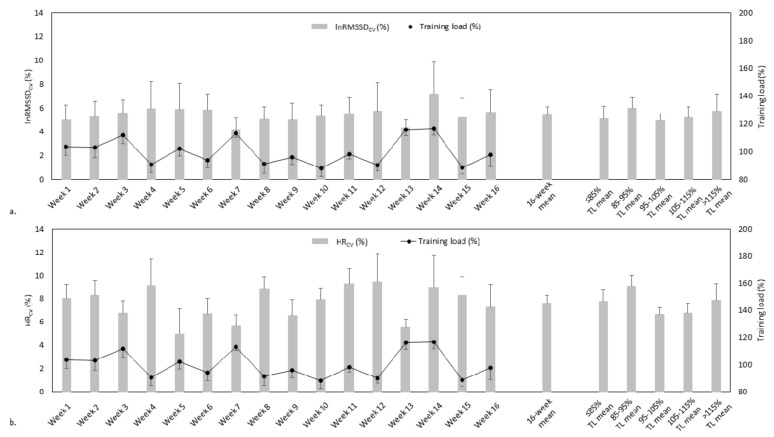
Weekly, 16-week mean, ≤85% training load mean, 85–95% training load mean, 95–105% training load mean, 105–115% training load mean and >115% training load mean for (**a**) lnRMSSD_CV_ and (**b**) HR_CV_. Data are mean ± 95% confidence interval. *n* = 11. HR, heart rate; HR_CV_, coefficient of variation in heart rate; lnRMSSD, natural logarithm of the root mean square of successive RR interval differences; lnRMSSD_CV_, coefficient of variation in natural logarithm of the root mean square of successive RR interval differences; TL, training load.

Figure 1a depicts the various reporting approaches used in lnRMSSD_CV_. The weekly lnRMSSD_CV_ ranged between 4.2 ± 1.0% (mean ± 95% confidence intervals) and 7.2 ± 2.7% across the 16-week recording period, while the mean weekly lnRMSSD_CV_ was 5.4 ± 0.7%. The mean lnRMSSD_CV_ ranged between 5.0 ± 0.6% and 6.0 ± 0.9% when categorised by percent training load, and was 5.0 ± 0.6% during weekly training loads of 95–105% of mean load.

Figure 1b depicts the various reporting approaches in HR_CV_. The weekly HR_CV_ ranged between 5.0 ± 1.9% and 9.5 ± 4.5% across the 16-week recording period, while the mean weekly HR_CV_ was 7.6 ± 1.3%. The mean HR_CV_ ranged between 6.7 ± 0.5% and 9.1 ± 0.9% when categorised by percent training load, and was 6.7 ± 0.5% during weekly training loads of 95–105% of mean load.

## 4. Discussion

The primary aim of this study was to determine the typical day-to-day variability in WHOOP3.0-derived HR and HRV in Olympic water polo athletes during a period of habitual training. Additionally, the impact of training load on day-to-day variability in WHOOP3.0-derived HR and HRV was determined, with a specific focus on determining day-to-day variability during weeks corresponding to typical/baseline training load. The primary finding was that the typical day-to-day variability in WHOOP3.0-derived HR and HRV is comparable to that of other recording devices and protocols reported in the scientific literature [2,13,14,15,16,17,18,19,20], regardless of training load. Consequently, the assessment of HR and HRV by WHOOP3.0 does not introduce any more variability than that which is naturally present in these variables.

The present study demonstrated typical day-to-day variability in WHOOP3.0-derived lnRMSSD of 5.4 ± 0.7%, ranging between 4.2 ± 1.0% and 7.2 ± 2.7% across individual weeks of the 16-week recording period (Figure 1a). Additionally, the day-to-day variability ranged from 5.0 ± 0.6% to 6.0 ± 0.9% when categorised for weekly training load as percentages of mean 16-week training load, and was 5.0 ± 0.6% during weekly training loads of 95–105% of mean load (Figure 1a). The typical day-to-day variability in lnRMSSD ranges between 3 and 13% [2,13,14,15,16,17,18,19], and this range is likely explained by subtle differences in assessment protocols, namely, the timing of assessment (i.e., morning waking versus nocturnal), posture (i.e., supine versus sitting versus standing), recording device and training load exposure at the time of assessment (i.e., no training/minimal training versus typical/baseline training versus overload training). Since the WHOOP3.0-derived HRV was obtained during overnight sleep periods in the present study, specific regard should also be given to the typical day-to-day variability in overnight sleep-derived lnRMSSD, where Costa et al. [18] demonstrated 4–6% variability using a Firstbeat Bodyguard electrocardiogram device. Given that the level of day-to-day variability in WHOOP3.0-derived lnRMSSD is comparable to that of other recording devices and assessment protocols, the present study indicates that the assessment of HRV by WHOOP3.0 does not introduce variability above that which exists organically. This finding, along with the acceptable level of validity in WHOOP3.0-derived HRV previously demonstrated [22], indicates that sport and exercise science practitioners may confidently utilise WHOOP3.0 to record HRV in practical settings.

The practical applicability of WHOOP3.0-derived HRV is further supported by contextualisation of the signal-to-noise ratio in the day-to-day variability in WHOOP3.0-derived lnRMSSD. Specifically, studies of physiological responses to acute training sessions indicate 10–20% changes in lnRMSSD [3,4], while a systematic review by Bellenger et al. [5] indicated 7–45% changes in lnRMSSD following chronic training interventions facilitating improved exercise performance. Together, the results of these studies indicate that utilising WHOOP3.0-derived HRV in practical settings would not limit the identification of a true physiological change in HRV, since the signal in HRV (i.e., the physiological change) is greater than the noise (i.e., the inherent day-to-day variability) that would be introduced by utilising WHOOP3.0-derived HRV.

Similarly to WHOOP3.0-derived lnRMSSD, the day-to-day variability in WHOOP3.0-derived HR (i.e., 16-week mean = 7.6 ± 1.3%; range = 5.0 ± 1.9% to 9.5 ± 4.5% across individual weeks of the 16-week recording period; range = 6.7 ± 0.5% to 9.1 ± 0.9% when categorised for training load; Figure 1b) is comparable to that reported in the scientific literature (~10–11%) [19,20]. Consequently, the variability in measures of HR introduced by WHOOP3.0 derivation is acceptable, and thus WHOOP3.0 may also be confidently utilised to record HR in practical settings.

While the typical day-to-day variability in WHOOP3.0-derived HR and HRV was quantified in a specific group of Olympic-level water polo players, the authors are confident that the results are generalisable to wider athletic populations. Specifically, the 3 to 13% range in day-to-day variability demonstrated in alternative HRV assessment protocols was captured over a range of team [2,13,15,16,17,18,19] and endurance [2,14] sports, with no evidence to suggest that day-to-day variability was impacted by sport.

The present study quantifies the typical day-to-day variability in WHOOP-derived HR and HRV using the manufacturer’s WHOOP 3.0 unit. Given that the proprietary hardware, algorithms and analytical methods of wearable technology are constantly evolving, future research should quantify the typical day-to-day variability of HR and HRV measured by contemporary WHOOP straps.

While WHOOP3.0-derived HR and HRV have been demonstrated to be *statistically* valid [22] and reliable (by the present study), it does need to be acknowledged that the *physiological* validity of using WHOOP-derived HR and HRV for inferring readiness to perform exercise remains unknown. Consequently, future research should determine the sensitivity of WHOOP-derived HR and HRV to acute and chronic changes in training load and exercise performance, and whether WHOOP-derived HRV may be used to individually guide training as has been shown using alternative assessment protocols and devices [9,10,11].

By means of its automated assessment of HR and HRV (i.e., by photoplethysmography during overnight sleep), WHOOP allows for frequent and convenient measurement of HR and HRV, and therefore enhanced application in athletes. Consequently, practitioners may be inclined to utilise WHOOP-derived HR and HRV in place of an existing recording device, but should do so with caution. Given the nuances in HRV assessment protocols (i.e., timing of assessment, posture, recording device, etc.), differences in absolute values of HRV and typical day-to-day variability in HRV are likely to exist between assessment protocols, and thus comparisons of longitudinal day-to-day changes in HRV between assessment protocols should be interpreted with appropriate caution.

In an attempt to evaluate the impact of training load on the day-to-day variability in WHOOP3.0-derived HR and HRV, the present study utilised WHOOP’s daily “Strain” metric as a measure of training load. However, the validity of this metric is presently unknown, and thus the training load categorisation analysis of day-to-day variability in WHOOP3.0-derived HR and HRV should be interpreted with appropriate caution. Future research should evaluate the validity of WHOOP Strain.

## 5. Conclusions

WHOOP3.0-derived HR and HRV demonstrate typical day-to-day variability of ~7.5% and ~5.5%, respectively. The contextualisation of this variability via the day-to-day variability in alternative HR and HRV assessment protocols and the signal-to-noise ratio indicates that the level of variability in WHOOP3.0-derived HR and HRV is acceptable for the inference of readiness to perform exercise in water polo players, and wider athletic populations. Given its capacity to measure HR and HRV via photoplethysmography during overnight sleep periods, WHOOP offers a convenient method of HR and HRV assessment, and its acceptable level of validity [22] and reliability allow it to be confidently utilised by sport and exercise science practitioners to record HR and HRV in practical settings.

## Data Availability

The datasets generated from the current study are available from the corresponding author on reasonable request.
